# Ascorbic Acid Inhibits Development of Tolerance and Dependence to Opiates in Mice: Possible Glutamatergic or Dopaminergic Modulation

**DOI:** 10.4103/0250-474X.40332

**Published:** 2008

**Authors:** S. K. Kulkarni, C. Deshpande, A. Dhir

**Affiliations:** Pharmacology Division, University Institute of Pharmaceutical Sciences, Panjab University, Chandigarh - 160 014, India

**Keywords:** Ascorbic acid, tolerance, dependence, dopamine, glutamate

## Abstract

In a recent study, it has been demonstrated that ascorbic acid possessed antidopaminergic activity and modulate the glutamatergic neurotransmission in mice. With this background, the present study was undertaken to study the effect of ascorbic acid on the development of tolerance and dependence to opiate and its mechanism of action. Male Swiss mice weighing 20-25 g were used in the present study. Mice were made physically dependent on opioid by the chronic administration of morphine (10 mg/kg, twice a day, for 9 days) intraperitoneally. Ascorbic acid, haloperidol (dopamine antagonist) or MK 801 (NMDA receptor antagonist) was administered daily for 9 d before challenging the animals with morphine. The development of tolerance was assessed by noting the tail-flick latency on day 1, 3, 9 and 10. On the 10^th^ day after the measurement of tail-flick latency, animals were challenged with naloxone (2 mg/kg., i.p.) and incidence of escape jumps were recorded by placing the animals in 45 cm high plexiglass container. Ascorbic acid (400-1600 mg/kg) dose dependently inhibited development of tolerance and dependence to morphine as noted from tail-flick latency. When given along with MK 801 (0.01 mg/kg., i.p) or haloperidol (0.1 mg/kg i.p.), ascorbic acid (800 mg/kg., i.p.) potentiated the response of MK 801 or haloperidol. In conclusion, it is hypothesized that inhibition of development of tolerance and dependence to morphine by ascorbic acid appears to have two components, namely dopaminergic and glutamatergic.

Various reports have confirmed the involvement of excitatory amino acids (EAA) in morphine tolerance and dependence^1^. Non-competitive NMDA receptor antagonist (like MK 801) and competitive NMDA receptor antagonist (like LY 274614) blocked morphine tolerance and dependence in rats^2^,^3^. Besides, in morphine tolerant and abstinent rats, binding of [^3^H] MK 801 in the presence of glutamate and glycine is decreased in some brain regions^4^,^5^, however, in the absence of glutamate and glycine, binding of [^3^H] MK 801 decreased modestly only in the cortex^6^. These data suggest that activities of endogenous glutamate and glycine are perhaps altered following activation of NMDA receptors in chronic morphine- treated animals. Ascorbic acid (AA) is shown to regulate NMDA receptor activity. Through the redox phenomena, AA has inhibitory role on NMDA receptor functioning^7^. Also, a growing body of research indicates that ascorbic acid is capable of modulating the effects of dopamine in the mammalian forebrain. Ascorbic acid enhanced the effect of catalepsy produced by dopaminergic antagonists as well as various nitric oxide synthase inhibitors^8^,^9^. Also ascorbic acid blocked the amphetamine-induced turning behaviours in rats with unilateral nigrostriatal lesion produced by 6-hydroxydopamine^10^. These findings coupled with inhibition of dopamineric agonists and antagonists in radioligand binding studies suggested antidopaminergic-like action of ascorbic acid. Recently, we have shown that ascorbic acid potentates the antipsychotic activity of both typical and atypical antipsychotics thus possessing antidopaminergic effect^9^. Thus, it was hypothesized that ascorbic acid has protective effect in the development of tolerance and dependence by modifying the dopaminergic or glutamatergic neurotransmission. With this background, ascorbic acid was tested for its ability to prevent the development of tolerance and dependence to opioids in mice and also to understand the possible mechanism of its action.

## MATERIALS AND METHODS

The following drugs were used: Ascorbic acid (S. D. Fine Chemicals, Mumbai, India), MK 801 [(+)-5-methyl-10,11-dihydroxy-5H-dibenzo(a,d)cyclo-hepten-5,10-imine], (Merck, Sharp and Dohme, England), haloperidol (Searle, Skokie, IL, USA), and morphine (Government Analytical Laboratory, Chandigarh). Haloperidol was dissolved in few drops of glacial acetic acid while morphine was dissolved in a few drops of dilute hydrochloric acid and volume made with distilled water. All the drugs were freshly prepared and administered intraperitoneally in a constant volume of 1 ml/100 g of body weight.

Male Swiss mice weighing between 20-25 g were obtained from the disease free small animal house, Hissar. The animals were housed under standard laboratory conditions and maintained on natural light and dark cycle and had free access to food and water. Animals were acclimatized to laboratory conditions before the experiment. Each animal was used only once. All the experiments were carried out between 0900 and 1500 h. The experimental protocols were approved by Institutional Animal Ethics Committee (IAEC) and conducted according to the Indian National Science Academy (INSA) Guidelines for the use and care of experimental animals.

### Experimental procedure:

The development of tolerance was assessed by tail-flick latency^11^ and as modified by Kulkarni^12^, while dependence was quantified by naloxone-precipitated withdrawal syndrome. The experimental protocol was the same as described by Kulkarni and Verma^13^. The animals were randomly divided into following groups: (i) saline: saline, (ii) saline: morphine (iii) ascorbic acid alone or in combination with other drugs (MK 801 or haloperidol): morphine (iv) ascorbic acid alone or in combination with other drugs (MK 801 or haloperidol). Ascorbic acid alone or with MK 801 or haloperidol was administered 30 min prior to morphine from days 1 to 9. Tail-flick latency was calculated on day 1, 3, 9 and 10. On day10, treatments were reversed such that, the animals that received, ascorbic acid alone or with MK 801 or haloperidol followed by morphine from day 1 to 9 were challenged with morphine alone and animals that received ascorbic acid alone or with MK 801 or haloperidol from day 1 to 9 were challenged with ascorbic acid alone or in combination with MK 801 or haloperidol followed by morphine. Immediately after the measurement of tail-flick latency, animals were challenged with naloxone (2 mg/kg., i.p.) and incidence of escape jumps were recorded in 45 cm high plexiglass container.

### Statistical analysis:

Data expressed as mean ± SEM was analyzed by one-way analysis of variance (ANOVA) followed by Dunnett's t-test. *p* < 0.05 was considered as statistically significant.

## RESULTS AND DISCUSSION

**Fig. 1:**
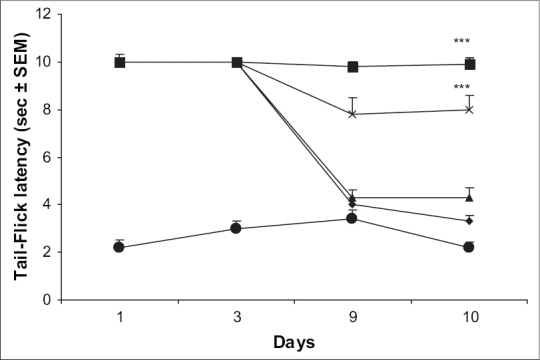
Ascorbic acid on the development of tolerance to the antinociceptive effect of morphine. Effect of chronic administration of morphine administration  on antinociceptive response and various doses of ascorbic acid (400-1600 mg/kg) on the development of tolerance to the analgesic effect of morphine was assessed employing tail-flick method in mice. *n* = 5-9 for various treatment groups. ***p < 0.001 as compared to morphine treated group. [–●– saline; –◆– Ctrl (morphine); –▲– AA (400); –×– AA (800); –■– AA (1600)].

Mice receiving chronic treatment with morphine (10 mg/kg, twice daily) showed maximal antinociceptive effect on days 1 and 3 of treatment. However, the animals showed rapid development of tolerance as the tail-flick latency time reached near the baseline latency by day 10. Mice treated with ascorbic acid (800 or 1600 mg/kg) followed by morphine (10 mg/kg) for 9 d showed considerable antinociception on day 1, 3 and 9 of testing. Challenging each group with saline followed by morphine on day 10 evoked considerable antinociceptive response. The effect was, however, not significant with low dose of AA (400 mg/kg) (fig. 1).

**Fig. 2:**
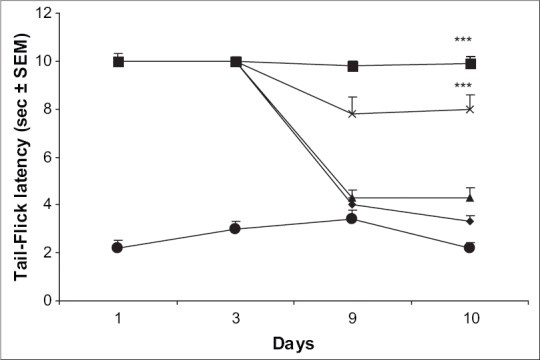
Effect of MK 801 alone and in combination with ascorbic acid on morphine tolerance. Effect of low doses of MK 801 (0.01 mg/kg) alone and in combination with ascorbic acid (800 mg/kg) to the analgesic effect of morphine was assessed employing tail-flick method in mice. *n* = 5-9 for various treatment  groups.  ***p < 0.001 as compared to morphine treated group. [–●– saline; –◆– Ctrl (Morphine); –×– AA (800); –▲– MK 801 (0.01); –■– AA(800) + MK 801 (0.01)].

Mice receiving MK 801 (0.01 mg/kg) followed by morphine (10 mg/kg) on days 1-9 displayed maximal antinociceptive effect on day 1 and 3 of treatment. However, the reaction time reached the baseline latency by day 9 of testing showing development of tolerance. On day 10, the tail-flick latency was no greater than that of control group receiving saline followed by morphine from day 1 to 10. However, mice pretreated with MK 801 (0.01 mg/kg) in combination with ascorbic acid (800 mg/kg) followed by morphine (10 mg/kg) on days 1-9 displayed significant antinociception throughout the testing period (fig. 2).

**Fig. 3:**
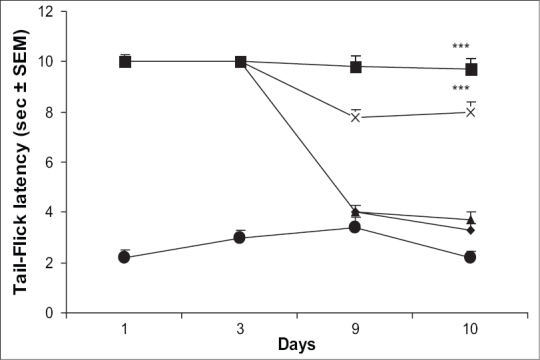
Effect of haloperidol alone and in combination with ascorbic acid on morphine tolerance. Effect of haloperidol (0.1 mg/kg) alone and in combination with ascorbic acid (800 mg/kg) on the development of tolerance to the analgesic effect of morphine was assessed by employing tail-flick method in mice. *n* = 5-9 for various treatment groups. ***p < 0.001 as compared to morphine treated group. [–●–saline; –◆– Ctrl (Morphine); –×–AA (800); –▲– Hal (0.1); –■– AA (800) + Hal (0.1)].

Mice receiving haloperidol (0.1 mg/kg) followed by morphine (10 mg/kg) on day 1-9 exhibited maximum antinociception on days 1 and 3. However, animals showed development of tolerance as the reaction time reached the basal latency by day 9 of testing. On day 10, when the mice were challenged with saline followed by morphine (10 mg/kg), exhibited tail-flick latency almost similar to that of control group receiving saline followed by morphine from day 1 to 10. However, animals receiving a combination of haloperidol (0.1 mg/kg) and ascorbic acid (800 mg/kg) on days 1-9 showed significant antinociception throughout testing period. Challenging the same group with saline followed by morphine on day 10 also evoked significant antinociceptive response (fig. 3).

**Fig. 4:**
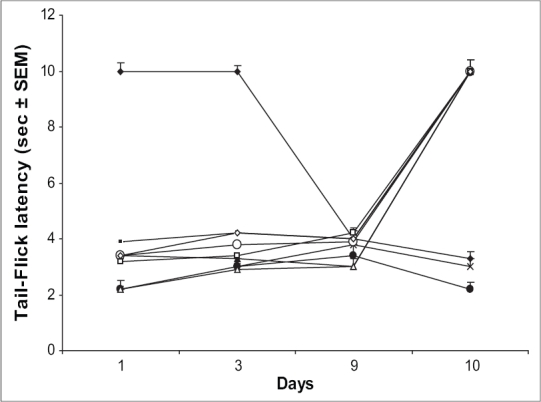
Effect of chronic treatment with ascorbic acid alone and in combination with haloperidol and MK 801 on analgesic response *Per se* effect of MK 801 (0.01 mg/kg) and haloperidol (0.1 mg/kg) alone or in combination with ascorbic acid (800 mg/kg) on analgesic response on different days. On 10^th^ day all the groups were challenged with respective treatment followed by morphine (10 mg/kg). (*n* = 5-9) for various treatment groups. [–●–saline; –◆– Ctrl (Morphine); –▲– AA (400); –×– AA (800); –■– AA (1600); –○– MK 801 (0.01); –△– AA (800) + MK 801; –□– Hal (0.1); –◊–AA (800) + Hal (0.1)].

Animals treated with ascorbic acid (800 mg/kg), haloperidol (0.1 mg/kg) or MK 801 (0.01 mg/kg) alone or in combination followed by saline failed to exhibit any significant antinociceptive response on days 1, 3 and 9. Challenging these groups with their respective treatments followed by morphine (10 mg/kg) on day 10 produced significant antinociception; a response similar to morphine effect on day 1 (fig. 4).

**Fig. 5:**
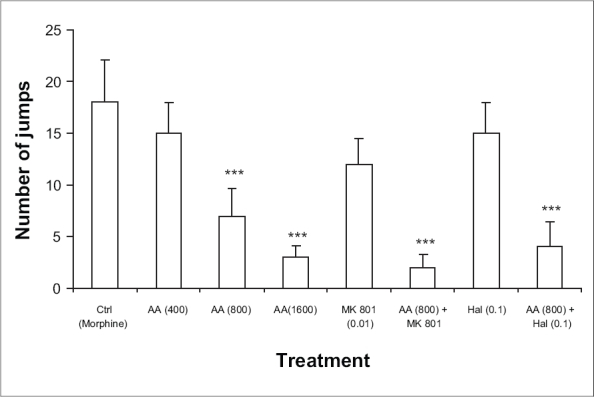
Effect of ascorbic acid alone or in combination with MK 801 and haloperidol on morphine withdrawal-induced jumps in mice. Effect of various doses of ascorbic acid (400-1600 mg/kg) and MK 801 (0.01 mg/kg) or haloperidol (0.1 mg/kg) alone or in combination with ascorbic acid (800 mg/kg) on naloxone-precipitated withdrawal jumps in morphine-dependent mice. (*n* = 5-9) for various treatment groups. ***p < 0.001 as compared to morphine treated group.

Mice receiving repeated treatment with saline followed by morphine (10 mg/kg) displayed numerous escape jumps in response to injection of naloxone (2 mg/kg) on day 10. Mice treated with ascorbic acid (400-1600 mg/kg) followed by morphine on day 1 to 9 dose-dependently inhibited the incidence of escape jumps. The effect, however, was not significant with ascorbic acid 400 mg/kg. Mice receiving MK 801 (0.01 mg/kg) followed by morphine (10 mg/kg) on day 1-9 failed to reduce naloxone-precipitated jumps significantly. However chronic treatment with haloperidol (0.1 mg/kg) alone and in combination with ascorbic acid (800 mg/kg) followed by morphine (10 mg/kg) from day 1-9 significantly decreased the naloxone-precipitated jumps. Similarly, mice receiving MK-801 (0.01 mg/kg) in combination with ascorbic acid (800 mg/kg) followed by morphine on day 1-9 significantly decreased the number of naloxone-precipitated jumps (fig. 5).

The development of physical dependence and tolerance with repeated use is a characteristic feature of all the opioid drugs and offer major limitation in their clinical use. Tolerance and dependence are thought to result from neuronal adaptations produced by repeated drug exposure^14^,^15^.

A series of reports have shown that non-competitive NMDA receptor antagonist, MK 801, blocks development of tolerance or dependence to several psychoactive substance including opiates^16^,^17^. The prevention of development of tolerance and dependence to opiates in our study appears to be due to different components of action of AA. The inability of low dose of ascorbic acid (400 mg/kg) given chronically to alter tolerance or dependence is consistent with observation that, ascorbic acid at low doses, has potentiating action on dopaminergic and glutamatergic system^9^,^18^. Ascorbic acid at high doses (800-1600 mg/kg), however, has profound inhibitory role over NMDA receptor function and a haloperidol-like antidopaminergic activity^7^,^19^. These activities might be acting simultaneously with the resultant inhibition of development of tolerance and dependence to morphine.

Dopamine antagonists like haloperidol and butaclamol inhibit wet-dog shakes and aggressive behavior observed during withdrawal^20^–^22^. Our findings showing inability of haloperidol to block development of tolerance may in part be due to low dose of haloperidol. However, ascorbic acid potentiated its antidopamineric action, as it does in stereotypy or catatonia^19^. Ascorbic acid inhibited the binding of both dopamine agonists and antagonists in dose dependent manner^23^–^25^. Furthermore, ascorbic acid also potentiated antidopaminergic activity of haloperidol^9^,^19^. Similarly, ascorbic acid significantly potentiated the activity of MK 801 that has no effect either on development of tolerance or dependence to opioids at low dose (0.01 mg/kg). Thus summing up the results, it appears that inhibition of development of tolerance and dependence by ascorbic acid has bi-directional component (or differential regulation). Since, even very high doses of ascorbic acid are known to be virtually non-toxic, it may be used therapeutically to treat morphine abstinence syndrome.

It is hypothesized that inhibition of development of tolerance and dependence to morphine by ascorbic acid appears to have two components, namely dopaminergic and glutamategic, and ascorbic acid prevents the development of tolerance and dependence to morphine in mice.
